# A Case of Notifiable Alithiatic Cholecystitis

**DOI:** 10.7759/cureus.54930

**Published:** 2024-02-26

**Authors:** Mariana Moniz Ramos, Inês Ambrioso, Frederica Ferreira, Maria M Grego, Marisa Brochado

**Affiliations:** 1 Internal Medicine, Hospital Distrital de Santarém, Santarém, PRT

**Keywords:** emergency department, infectious diseases, antibiotics, cholecystectomy, alithiatic cholecystitis, listeria monocytogenes

## Abstract

Acute alithiatic cholecystitis is an inflammation of the gallbladder without evidence of gallstones, often due to infection. It can occur at any age, and it is predominant in males.

Listeriosis is a rare bacterial infection caused by Listeria monocytogenes (LM) through the ingestion of contaminated food such as dairy, legumes, and raw meats. Clinical presentations of listeriosis include bacteremia, meningitis, and gastroenteritis. Acute cholecystitis caused by listeria is even more uncommon, with only 23 cases reported in the literature.

We present a case of a 65-year-old male, admitted to the Emergency Department with fever and altered state of consciousness which revealed bacteremia due to cholecystitis to LM. The patient was submitted to laparoscopic cholecystectomy and appropriate antibiotic coverage and was discharged seven days later. Early recognition and treatment of this disease are crucial for reducing morbidity and mortality.

## Introduction

Acute alithiatic cholecystitis is an inflammation of the gallbladder without evidence of gallstones, often due to infection. Listeria monocytogenes (LM) is Gram-positive bacteria, a facultative anaerobic pathogen involved in serious food-borne diseases, which is responsible for listeriosis. It mainly affects elderly patients, pregnant women, and immunocompromised people. Infection in immunocompetent patients is rare [1,2].

Although bile has antimicrobial properties by disrupting bacterial membranes, LM has evolved to survive in bile using bile salt hydrolases, which decreases bile toxicity and the bile exclusion system, thereby increasing tolerance to bile. Although it is known that LM can colonize the intestine and the bile, bile duct infections and acute cholecystitis are uncommon symptoms. [1].

Clinical presentations of listeriosis include bacteremia, meningitis, and gastroenteritis [3]. Cases of bacteremia complications in CNS listeriosis are relatively high, at about 40%, and are high-risk cases with a high mortality rate [4]. Penicillin, ampicillin, amoxicillin, and gentamicin are recommended for the treatment of listeria infections [5].

This article was previously presented as a meeting abstract at the Eighth National Urgency Meeting on October 8, 2023.

## Case presentation

A 65-year-old male, with a personal history of type 2 diabetes, treated with oral antidiabetic medication, was brought to the emergency department with fever (tympanic temperature 39.4ºC) and altered state of consciousness within 24 hours. The patient presented 10 points on the Glasgow Coma Scale and abdominal pain on generalized palpation. There were no neck difficulties or meningeal signs. 

**Figure 1:**
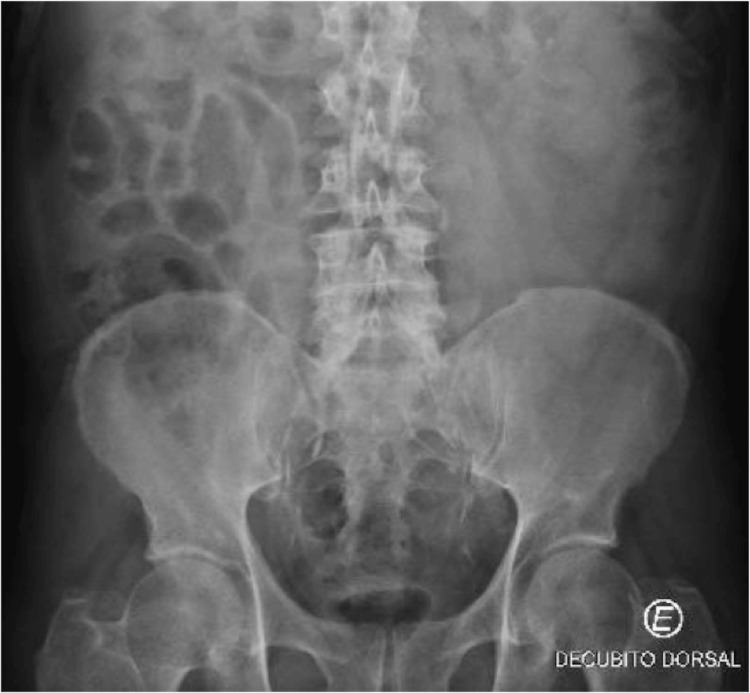
Abdominal x-ray with aerocolia

**Figure 2:**
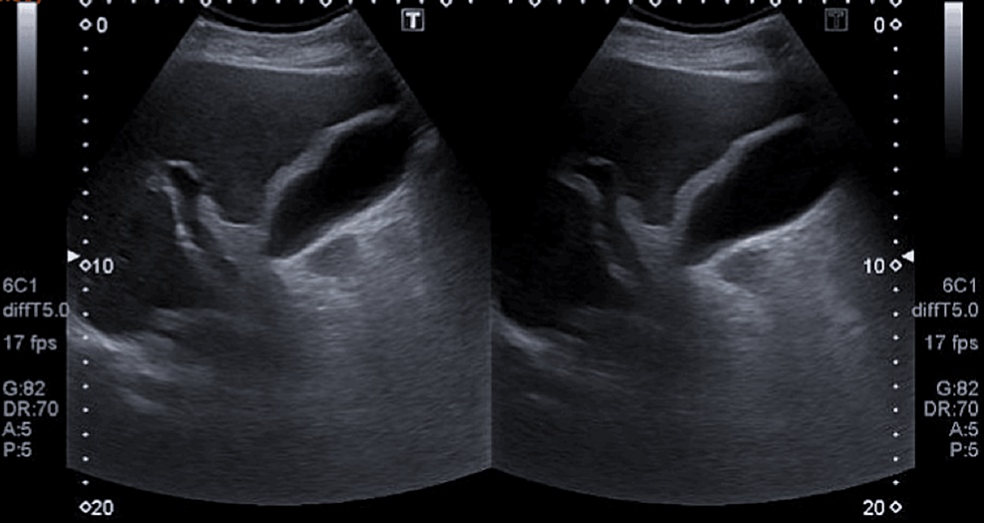
Abdominal ultrasound with a moderately distended gallbladder, admitting mild diffuse parietal thickening, without lithiasis or biliary sludge, findings compatible with alithiasic cholecystitis

Of the complementary exams, the following stand out: analytically hemoglobin 13.4 g/dL; white blood cell counts 11.2x10E9/L with 81.1% neutrophilia; platelets 140x10E9/L; INR 1.26; Urea 55 mg/dL; Creatinine 1.4 mg/dL; K 3.0 mEq/L; Total bilirubin 1.7 mg/dL; Direct bilirubin 0.7 mg/dL; AST 68 U/L; Alt 55 U/L; LDH 273 U/L; GamaGT 137 U/L; CK 1,037 U/L; Amylase 139 U/L; PCR 5.80 mg/dL, negative antigenuria, innocent urine II; CT-CE and chest x-ray were unremarkable; abdominal x-ray with aerocolia (Figure 1) and abdominal ultrasound with a moderately distended gallbladder, admitting mild diffuse parietal thickening, without lithiasis or biliary sludge - findings compatible with alithiasic cholecystitis (Figure 2).

**Figure 3:**
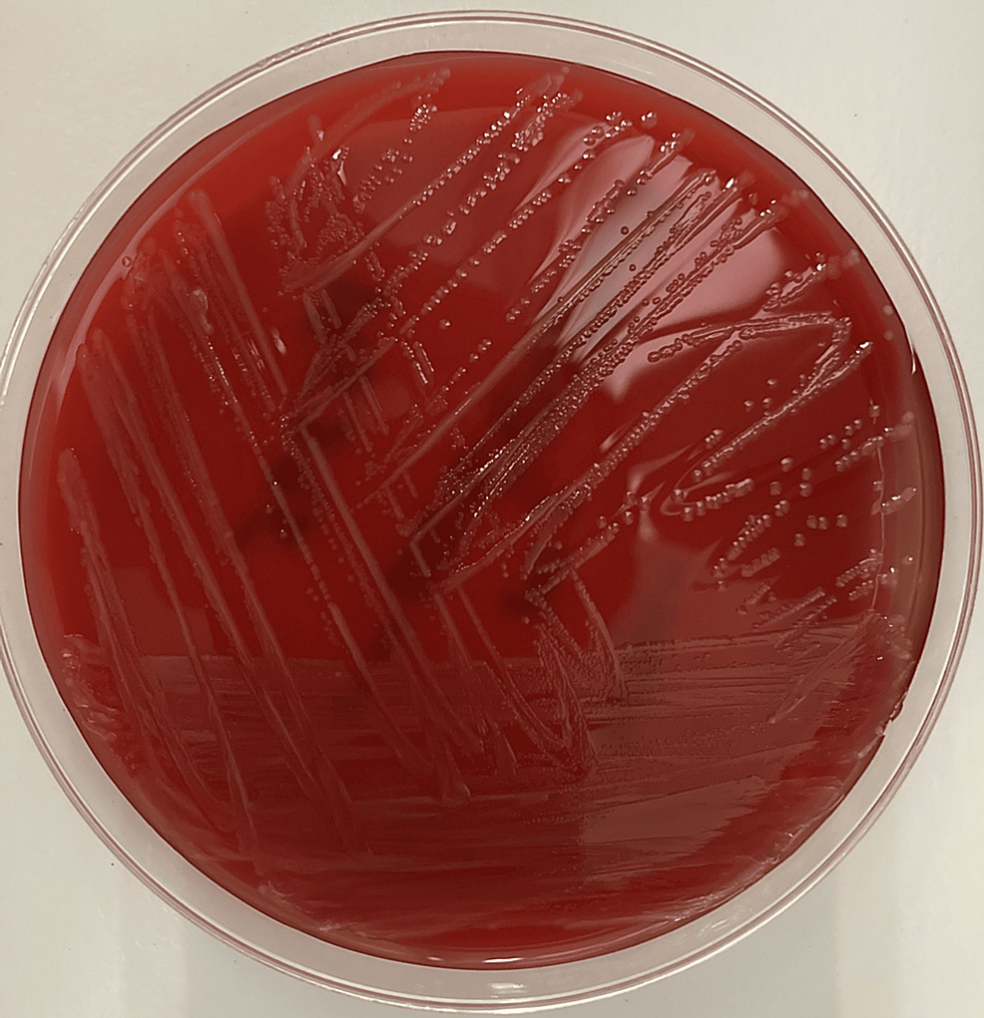
Isolation of listeria monocytogenes from blood cultures

He was submitted to laparoscopic cholecystectomy and received broad-spectrum antibiotic therapy with piperacillin/tazobactam for seven days. Amoxicillin-sensitive LM was isolated from the microbiological study of the bile and from blood cultures (Figure 3). Antibiotic therapy was switched to amoxicillin/clavulanic acid for another seven days and the patient was discharged.

## Discussion

Acute cholecystitis caused by listeria is extremely rare [6]. Listeriosis is a mandatory notifiable disease. It can affect everyone but immunocompromised patients, newborns, the elderly, and pregnant women are patients at higher risk. Symptoms vary depending on the organ system affected. Gastroenteritis is common after ingestion of contaminated food. Less commonly, LM can cause a focal site of infection such as acute cholecystitis [7-9].

Listeria sepsis or bacteremia accounts for one-third of adult cases of invasive listeriosis, with symptoms typically manifesting as fever and chills [4]. Listeria infections are diagnosed through blood or cerebrospinal fluid cultures. In all Listeria infections, the highest titers of immunoglobulin G (IgG) agglutinins are found two to four weeks after the onset of illness [8,10].

Penicillin, ampicillin, amoxicillin, and gentamicin are recommended for the treatment of Listeria infections [4]. Cephalosporins have no in vitro activity and should not be used; failures with vancomycin have been reported. Linezolid is active in vitro, but there is insufficient clinical experience [4,5].

For individuals who report allergies to penicillin and betalactams, trimethoprim/sulfamethoxazole is an effective alternative. In the current case, our patient was empirically treated with piperacillin/tazobactam on his initial presentation after cholecystectomy, which covers LM, and early coverage of LM may have contributed to his survival.

## Conclusions

The authors describe a rare case of listeriosis due to cholecystitis in a diabetic patient. Listeriosis is a rare pathology, occurring in only 2%-15% of all cases of acute cholecystitis and the majority of affected patients are immunocompromised. However, in around 25% of cases, it occurs in apparently healthy patients over 60 years of age. This case highlights the need to pay attention to all possible infectious foci, especially in diabetic patients.
